# Radio Tracking Reveals the Home Range and Activity Patterns of Nutria (*Myocastor coypus*) in the Macdo Wetland in South Korea

**DOI:** 10.3390/ani13101716

**Published:** 2023-05-22

**Authors:** Maniram Banjade, Pradeep Adhikari, Sun-Hee Hong, Do-Hun Lee

**Affiliations:** 1 National Institute of Ecology, 1210 Geumgang-ro, Maseru-myeon, Seocheon-gun 33657, Republic of Korea; mani88zoo@gmail.com; 2 Institute of Humanities and Ecology Consensus Resilience Lab, Hankyong National University, Anseong 17579, Republic of Korea; pdp2042@gmail.com (P.A.); shhong@hknu.ac.kr (S.-H.H.)

**Keywords:** behavior, introduced, Macdo wetland, nutria, radio tracking

## Abstract

**Simple Summary:**

This study aims to investigate the behavior and ecology of nutria (*Myocastor coypus*), a semi-aquatic rodent that was introduced to South Korea for commercial farming and subsequently damaged aquatic ecosystems after its release. How the behavioral ecology of introduced nutria changes over time and across seasons remains unclear. Twenty-four adult nutria (twelve males and twelve females) were radio tracked in 2015–2016 to identify their home range size and activity patterns in the Macdo wetland, South Korea. This study found that the nutria home range size varied seasonally, with males having larger home ranges than females. Additionally, nutria showed crepuscular and nocturnal activity patterns throughout the year, with no significant difference between sexes. The findings of this study provide crucial information on the home range and activity patterns of introduced nutria in the Macdo wetland, which can guide management efforts to mitigate their impacts on the ecosystem. It is the first quantitative analysis of the home range and activity patterns of introduced nutria based on radio tracking data in the Macdo wetland.

**Abstract:**

Nutria (*Myocastor coypus*) are semi-aquatic rodents that were introduced in South Korea for commercial farming but significantly damaged aquatic ecosystems. Understanding nutria ecological behavior is essential for developing effective control and eradication strategies to mitigate their impacts. Thus, this study aimed to investigate the home range and activity patterns of 24 nutria (12 males and 12 females) in the Macdo wetland in South Korea from 2015–2016 through radio tracking. The average minimum convex polygon home range of the nutria was 0.29 ± 0.55 km^2^, with a 95% kernel density estimation (KDE) home range of 0.43 ± 0.85 km^2^ and a 50% KDE home range of 0.05 ± 1.1 km^2^. The home range of males was larger than that of females; however, the winter home range of females was as large as that of males. The home range also varied seasonally, with the smallest observed in winter. The nutria showed crepuscular and nocturnal activity patterns throughout the year, with no significant difference between sexes. The activities in spring, summer, and autumn showed no significant differences, but the activity in winter was significantly different from that in the other seasons. This study may serve as a basis for developing appropriately timed and scaled management strategies to mitigate the impacts of nutria on ecosystems. In conclusion, several environmental and biological factors contribute to the behavior of nutria in South Korea.

## 1. Introduction

Invasive alien species are a major contributor to the current global biodiversity crisis, impacting native biodiversity communities through competition, ecological changes, economic costs, and health risks [1,2]. Among invasive vertebrates, nutria (*Myocastor coypus*) are semi-aquatic rodents native to subtropical and temperate regions of South America that have now spread to every continent except Australia and Antarctica. They are used for meat and fur production [3,4,5]. Non-native nutria has caused substantial ecological and economic damage by destroying cultivated crops, aquatic vegetation, and trees. Hence, the nutria is listed as one of the 100 most invasive alien species by the International Union for Conservation of Nature [3,6].

Nutria were introduced to South Korea in 1985 to boost the local economy through breeding farms; however, the demand for nutria products did not meet expectations, causing a decline in the farming business [6]. Consequently, nutria, like many other mammalian species, were intentionally released or escaped into the natural environment, establishing populations in several areas, including Jeju Island [7,8]. By 2014, they were present in 19 administrative districts, posing threats to agricultural and irrigation systems because of aggressive foraging and burrowing [9]. Nutria also have a negative impact on public health as they transmit zoonotic diseases, such as leptospirosis and toxoplasmosis [10,11].

Nutria are difficult to eradicate because of their high fecundity [12]. Lethal control through trapping and hunting has been effective at the local level in Britain, the Delmarva Peninsula in the United States, and South Korea [3,13]. In response to public requests regarding increased agricultural damage, the Korean government launched a 5-year “Nutria Eradication Project,” in which 27,487 nutria were captured between 2014 and 2018 [6]. Although their distribution range has been reduced to 14 local administrative districts, nutria persist in the Nakdong River Basin [9]. If effective control measures are not implemented, the nutria population may spread further to earlier distribution sites and across the entire nation.

Effective management of nutria requires a comprehensive understanding of their ecology and behavior [14]. Specifically, their home range and activity patterns must be assessed to develop suitable control and eradication methods and to optimize the spatial and temporal aspects of control initiatives. Very-high-frequency (VHF)-based radio tracking systems are widely used to monitor nutria [15] owing to their low operating cost and high efficiency and their ability to be detected under water [16].

Several studies have focused on the ecology, diet, distribution, climate change-induced habitat suitability, and impact of nutria on agriculture and aquatic ecosystems [8,9,17,18]. However, a detailed investigation of their home range and activity patterns remains lacking to date. Thus, this study aimed to evaluate the home range and activity patterns of nutria according to sex and season by using a VHF-based radio tracking system. The results of this study are expected to provide valuable information to the government regarding the legislation of management policies that can effectively control and eradicate nutria at federal and national scales.

## 2. Materials and Methods

### 2.1. Study Site

**Figure 1:**
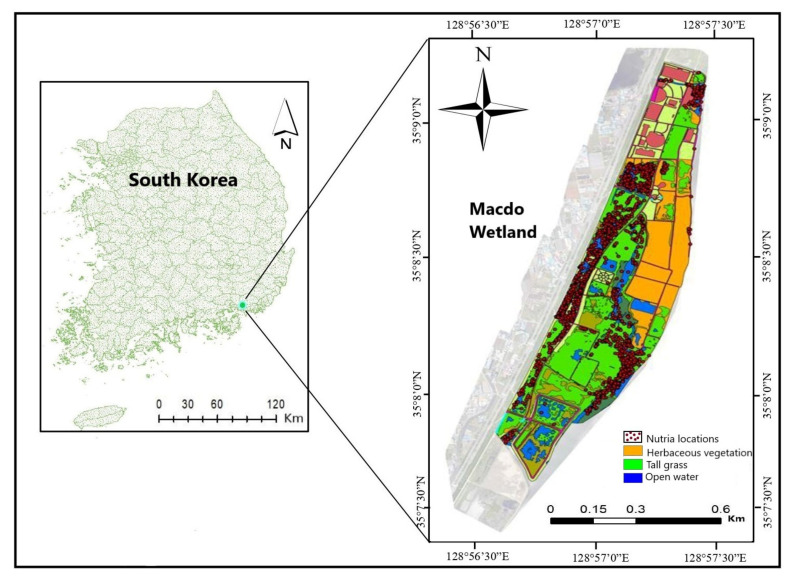
Map showing the Macdo wetland and its location in South Korea. Spatial habitat types in the Macdo wetland and the movement tracks of 24 nutria individuals in 2015–2016 are shown.

The Macdo wetland (126°34′30″–126°39′00″ E, 37°15′00″–37°160′30″ N) is situated in Busan Metropolitan City in the southeast corner of the Korean Peninsula. It covers a rectangular area of 2.58 km^2^ with a length of 6.90 km (Figure 1). The lowest and highest monthly mean temperatures at the study area, based on Busan weather station data, are −3 °C and 25 °C, respectively. The primary vegetation types in this region include *Bromus japonicus*, *Alisma orientale*, *Setaria faberi*, and *Oenanthe javanica*. This wetland is one of Korea’s most representative seasonal habitats for migratory birds, making it a haven for bird watchers. The wetland is also an important nutria habitat, with records dating back to 2009 [6].

### 2.2. Capturing and Handling

**Figure 2:**
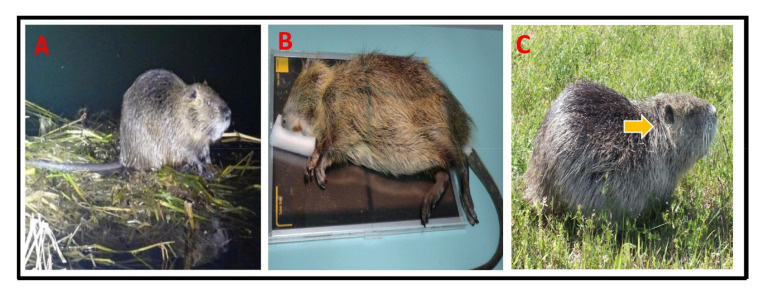
Overview of nutria activity in the Macdo wetland. (**A**) Nocturnal sighting of a nutria individual, (**B**) assessment of captured individuals, (**C**) nutria with a radio transmitter (arrow pointing to transmitter around its neck).

Live traps were placed in areas with nutria activity in October 2015, with apples and carrots used as bait. The traps were checked daily. Captured nutria were anesthetized with alfaxalone (0.4 mL/kg) administrated intravenously, and basic information such as sex and weight was recorded. A matched sample of 24 adult nutria (12 males and 12 females) were selected for this study. Each nutria was fitted with a VHF radio transmitter (R2030, ATS Inc., Minneapolis, MN, USA) attached to its neck and released at a site 100 m from the capture point (Figure 2).

One week after release, the individuals were radio tracked using a truck with a VHF radio collar antenna (ATS Inc., Minneapolis, MN, USA) attached to a handheld receiver (ICOM Inc., Osaka, Japan) to obtain details regarding their movement and activity patterns. The location of each individual was verified by direct observation after homing in on the signal. The survey was conducted once a month for three consecutive days and nights, and the data collected included GPS positions, such as latitude, longitude, date, and time. The strongest signals were used to access the individual to minimize bias. The ethical guidelines published by American society of mammologist for conducting research involving animals was followed [19]. During the survey period, 12 surveys were conducted, and the number of locations varied from 12 to 195 owing to field conditions and individual behavior variability.

### 2.3. Data Analysis

#### Home Range Estimation

When estimating the home range of each individual, we considered only locations with statistical independence based on the Schoener index [20]. We estimated the home range of each nutria based on 100% minimum convex polygon (MCP), 95% fixed-kernel density estimation (KDE_95_), and 50% KDE (KDE_50_) [21,22] by using the Home Range Tool extension in ESRI ArcMap 10.1 (ESRI, Redlands, CA, USA). MCP is a simple method often criticized for overestimating home range [23]. KDE utilizes a nonparametric probability density function to account for the nonlinear curved outlines of the home range [24]. KDE_50_ reflected the core area of the home range of each nutria. The least-squares cross-validation estimator was used to calculate the smoothing factors for the fixed-kernel estimators [25]. We compared the average and seasonal home range sizes between sexes using Welch’s *t*-test [26], whereas analysis of variance was used to compare seasonal differences in the average home range. The chosen p-value for significance was set at *p* < 0.05. 

### 2.4. Activity Patterns

To determine the activity patterns of the studied species, we considered the locations obtained during sampling at 1–2 h intervals for three consecutive days and nights each month. We then evaluated differences in the probability of a nutria being active (each nutria location was binary coded: 1 = active, 0 = inactive) in relation to the period of the day, sex, and season. Periods were defined by sunlight: diurnal, activity during the day; nocturnal, activity at night; crepuscular, activity during twilight; and cathemeral, no differences in activity between the three time periods [27]. The Wald chi-square test was used to examine differences in the probability of a nutria being active in relation to the period of the day, sex, and season.

## 3. Results

### 3.1. Home Range of Nutria

**Figure 3:**
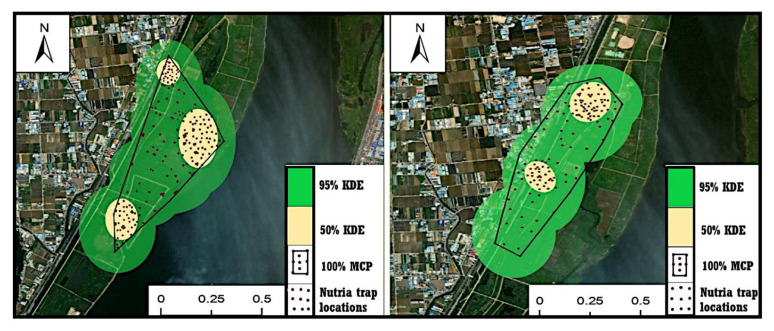
Minimum convex polygon (MCP) and kernel density estimation (KDE) home range of two nutrias (ID Nos. 1 and 3) in the Macdo wetland, South Korea.

Between October 2015 and September 2016, we recorded 2336 independent locations from the 23 adult nutrias (11 males and 12 females) fitted with radio transmitters (Table S1). The transmitter of nutria #N18 was destroyed; thus, its activity was not monitored. However, the data from 23 individuals were monitored sequentially throughout the survey period. During the 12-month survey period, we estimated the home ranges of individuals in 12–195 locations. The 100% MCP analysis revealed an average home range of 0.29 ± 0.55 km^2^ (range 0.03–0.88 km^2^) over the entire tracking period. The mean KDE_95_ and KDE_50_ were 0.43 ± 0.85 km^2^ (range 0.01–0.71 km^2^) and 0.05 ± 1.1 km^2^ (range 0.004–0.18 km^2^), respectively (Table 1). No significant difference in seasonal home range was found between males and females in winter (*t* = 2.29, *p* = 0.064), and the winter home range estimated by KDE_95_ was similar for both sexes (Table 1). However, when the entire study period was considered, males had a significantly larger home range than females (*t*-test, *t* = 2.26, *p* = 0.03; see Table 1). Nutria home range showed seasonal changes regardless of the estimator used (MCP = 3.31, *p* = 0.021; KDE_95_ = 2.64, *p* = 0.006; KDE_50_ = 3.80, *p* = 0.005). The smallest nutria home range was observed in winter among all seasons (Table 1). Typical home ranges (MCP and KDE) for nutria #N1 and #N3 are shown in Figure 3. A summary of the seasonal home ranges for each sex and their test statistics are presented in Table 1.

### 3.2. Activity Patterns of Nutria

**Figure 4:**
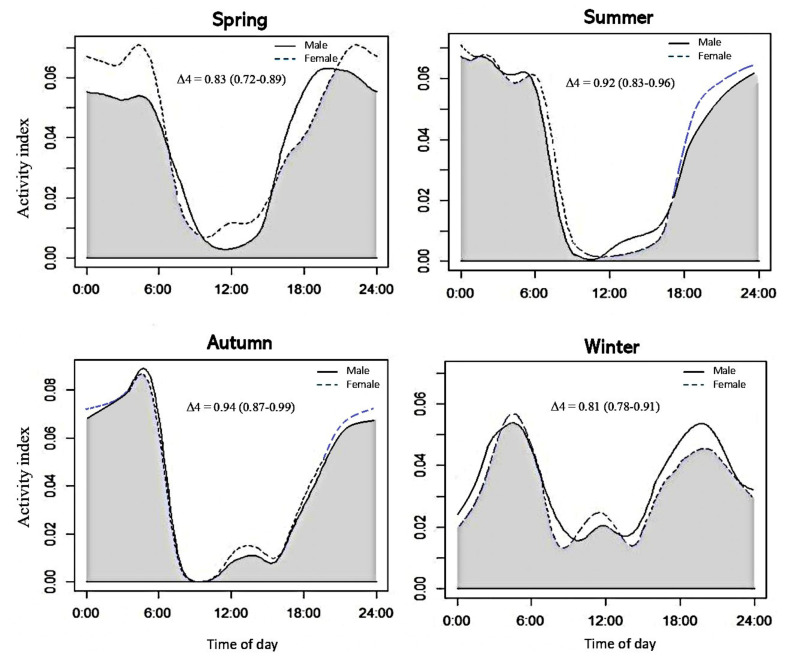
Seasonal activity patterns of male and female nutria in the Macdo wetland. The activity patterns during winter differ from those observed during the other seasons.

When the entire period was considered, the nutria showed unimodal crepuscular activity (with approximately 39.6% of locations at dawn and dusk) and nocturnal activity (on average, 46.2% of locations were at night) peaks (Figure 4). Less than 14% of all nutria locations were recorded between 06:00 and 18:00, indicating minimal diurnal activity. Nutria activity significantly varied along the circadian period, with the highest activity during twilight and the lowest activity during the day (χ^2^ = 10.02, *p* = 0.015). We did not detect different activity patterns between sexes (χ^2^ = 3.21, *p* = 0.06), but females had greater activity intensity in almost every season than males (Figure 4). No significant difference in nutria activity was observed between spring, summer, and autumn; however, the activity in winter was significantly different from that in the other seasons (Table 2). Although winter had the lowest number of nutria trap locations (*n* = 252), the activity curve displayed evidence of activity during certain times of the day.

## 4. Discussion

An in-depth understanding of ecological behavior is essential for effectively managing alien and invasive mammals that have been successfully introduced, established, and spread in new environments [28]. The behavior of such species is influenced by various factors, including local environmental factors, such as habitat size and habitat type; biological factors, such as capture and killing; and physical factors, such as dams and walls in the area they inhabit [29]. However, specific behavioral information on nutria in South Korea is currently unavailable. Even within the introduced range, such information is scarce and often only available in the native Korean language. This is partly due to the difficulty in continuously recording individual behavior in the field, considering that these animals spend most, if not all, of their time in or near aquatic habitats. 

This study utilized radio telemetry to extract behavioral data on the home range and activity patterns of nutria, aiming to guide the implementation of the nutria eradication project. In the present study, the average MCP home range was 0.29 ± 0.55 km^2^, and the KDE_95_ and KDE_50_ were 0.43 ± 0.85 and 0.05 ± 1.1 km^2^, respectively. The range observed in our study is similar to their native South American range [30], but larger than that (95% MCP of 0.043 km^2^, KDE_95_ of 0.085 km^2^, and KDE_50_ of 0.018 km^2^) in similar habitats along the Miryang wetland in South Korea [31]. This difference in home range may be associated with the available habitat. For instance, home ranges are smaller in small wetlands and larger in large wetlands. The Miryang wetland is narrow (0.21 km^2^) and bounded by roads and fences, creating a hostile matrix that is difficult for nutria to travel across. By contrast, our study area is a broad riparian habitat (2.58 km^2^) adjacent to the Nakdong River. Food and adequate feeding and nesting platforms may spread over greater distances, resulting in a broader home range. Such large home ranges have also been observed for species inhabiting open areas [32].

Our study also revealed sex differences in home range, with male nutria exhibiting a home range that is 0.23 km^2^ larger than female nutria on average. Sex differences in home range have also been observed for nutria in introduced [31] and native ranges [30,33]. This phenomenon is consistent with the general trends observed in various rodents [34,35]. The observed differences between sexes are often attributed to differences in body size and, consequently, differences in their energy requirements. Because of the difference in energy requirements between sexes, males would cover larger foraging areas than females [36]. Significant differences in body weight between sexes were observed in the present study. Although nutria are not sexually dimorphic, males are heavier and larger than females [37], which could partly explain the observed differences in home range between sexes.

Males and females differ in reproductive behavior. Males are typically more aggressive and engage in more territorial behavior than females, creating hierarchies prior to the mating season [38] and mating with multiple females [12]. Reproductive behavior also influences differences in home range between sexes, with males searching larger areas for mates [39] and females reducing their home ranges to fulfill the energy needs of reproduction. In many species, the spatial organization of males is strongly influenced by the distribution of females, as the reproductive success of males is often determined by their ability to find and defend mates [40,41]. The mating system in nutria is a resource defense polygyny, wherein dominant males compete to monopolize key resources (territory and foraging areas) that are then used by reproductive females [12,42]. Therefore, by occupying a large home range, males can maximize their chances of mating and fertilize more females, leading to greater reproductive success [43].

The ecological functions of rodents are related to their activity and space use patterns, which may exhibit seasonal variations [44]. In the present study, the home ranges of nutria varied seasonally and were considerably smaller in winter than in the other seasons (Table 1). Winter survival necessitates physiological and behavioral modifications [45] because movement costs can be exceptionally high owing to low temperatures. Furthermore, the reduced availability of food resources during winter may account for small home ranges. In response to these challenges, rodents tend to minimize locomotion and spend more time in nests or resting areas to conserve energy, particularly during winter [46,47]. 

Nutria exhibit predominantly nocturnal and crepuscular activity patterns, with some individuals displaying diurnal activity during winter. The activity patterns of the nutria analyzed in the present study were similar to those of other nutria populations in Argentina [48], Italy [49], and South Korea [31]. Other similar competing species sometimes present similar circadian activity patterns, with increased activity during twilight and nighttime, although marked differences have been found between sexes [50]. For instance, Zschille et al. [50] found sex differences in American minks. Further comprehensive studies may reveal sex differences in nutria activity that were not evident in the data collected in this study.

In the present study, no seasonal differences in nutria activity patterns were found during spring, summer, or autumn. However, the activity in winter differed significantly from that in the three other seasons (Table 2). Small bouts of diurnal activity occurred in winter, when the night was colder, and nutria foraged during day. The local absence of wild predators or control plans may have favored the activity patterns of nutria during the day. This interpretation is supported by previous findings that nutria may become diurnal during cold months [51,52]. This adaptation to daylight hours is presumably a response that minimizes energy loss and maintains adequate food intake at low environmental temperatures [53]. The freezing of water surfaces at night can prevent nutria from accessing food sources, such as aquatic vegetation [54,55]. Accordingly, food provided to captive nutrias throughout the 24 h cycle restores the crepuscular and nocturnal behaviors of this large rodent [53,56].

Radio-tagged nutria in Germany are diurnal and never detected during crepuscular or nocturnal hours [57]. However, in our study area, nutria activity peaked after sunset and continued until sunrise. The highest activity during twilight and nighttime could be associated not only with rising temperatures during the day but also with stress conditions produced by human activity. Our study site is located adjacent to Busan (the second largest city in South Korea), which has a population of over 4316 inhabitants/km^2^. During the day, people frequently use this area and outdoor facilities, often in family groups or with dogs. This increased human activity during the day may restrict the activity of nutria, as has been proposed in suburban areas where they have been introduced [49]. Furthermore, retaliatory killing/capturing following the eradication program [6] has created a “landscape of fear” for nutria, most of which have adopted crepuscular and nocturnal behavior in our study area. Several individuals were captured/killed from 2014 to 2018 in South Korea (including our study sites), which might have increased nutria activity during the darkest hours. During the implementation of the nutria eradication project in South Korea, several individuals were caught or killed during daylight [6]. Considering this information, we predict that these diurnal hunters would have placed substantial pressure on nutria, leading to the evolution of crepuscular and nocturnal activity patterns to reduce their probability of encountering humans. Many species undergo rapid and significant behavioral changes owing to strong human harvest selection [58]. Although the hunting pressure is currently low for the nutria population under study, the temporal activity patterns that evolved in response to earlier selection pressures may still be present. 

Behavioral plasticity has been observed in nutria, which can adapt to a wide range of environmental conditions, including natural ponds, swamps, and riverine habitats. In South Korea, introduced nutria have successfully settled in the Nakdong River Basin [4]. Various studies have predicted that suitable habitats are confined to midstream and downstream riverine areas of basins that experience relatively mild winters [9,59]. As a result of ongoing global warming, temperatures in the basin are expected to increase, particularly in the upstream regions [60]. If proper control measures are not implemented, nutria will retain their current distribution, and further expansion is likely to occur gradually from south to north through the water channel [9]. Hence, eradication efforts should consider dividing this broad Nakdong River Basin into two distinct management zones (i.e., middle and lower Nakdong Basins) which can be treated as individual units and are large enough to minimize the chance of reinvasion from adjacent zones. Isolation of nutria populations is likely to improve the effectiveness of eradication efforts because it reduces adaptability through loss of genetic diversity or inbreeding, which can ultimately lead to extinction [61].

Poisoning, shooting, and trapping were traditionally employed to mitigate the impacts of nutria. In South Korea, the use of rodenticides is restricted because of their consumption hazards to non-target species, and shooting is prohibited. Trapping is commonly used for controlling nutria populations because of its low cost and effectiveness [13,62,63]. Considering that females are prolific breeders, trapping females within their home ranges should be considered as a reference to delineate control actions. Thus, any control plan that aims to reduce nutria populations in areas similar to our study area should involve placing traps on every kernel home range of 0.39 ± 0.2 km^2^. Furthermore, nutria activity peaked from dusk to dawn, suggesting that trapping to capture individuals for eradication may be most effective between 18:00 and 06:00. Multiple capture cage traps (MCTs) could be a good option for capturing nutria in urban and rural habitats. The ability of MCTs to capture multiple individuals of all size ranges is particularly important when dealing with species such as nutrias, which form social groups [64].

Our study focused on the Macdo wetland, which is only a small portion of the large Nakdong River Basin. The microhabitat of nutria could vary in different regions of the river and connected wetlands. The findings of this study may not fully represent the overall nutria habitats in South Korea. Therefore, further studies are required to better understand the activity pattern and ecological impact of nutria on the aquatic ecosystem. Future data collection efforts should focus on the use of microhabitats, mating/reproductive strategies, dietary preferences, and dietary item variations that may drive shifts in home ranges and activity patterns. The potential impacts of nutria control on nesting bird species and other native wildlife that may be present should also be considered.

## 5. Conclusions

Our study described the seasonal home ranges and activity patterns of nutria introduced in South Korea, and the results showed significant differences in home range size between sexes and seasons. Introduced nutria showed more intense activity during crepuscular and nocturnal hours and a relatively lower activity intensity in the afternoon. This study provides crucial information for controlling nutria in the study area and other regions with similar characteristics. It is the first quantitative analysis of the home range and activity patterns of introduced nutria based on radio tracking data in the predator-free Macdo wetland in South Korea. Future studies should consider investigating larger areas and take into account other variables that may impact the behavior of nutria in order to develop more comprehensive management strategies for controlling the ecological impact of nutria in South Korea and other regions. 

## Figures and Tables

**Table 1:** Seasonal home range (km^2^) by 100% minimum convex polygon (MCP) and 95% and 50% kernel densities (KDEs) at the study site.

Season	Method	Male (*n* = 11)	Female (*n* = 12)	Sex Difference	
				*t*	*p*
Spring (Mar–May)	MCP	0.37 ± 1.3	0.25 ± 2.4	1.81	0.043
	KDE_95_	0.51 ± 1.2	0.35 ± 3.2	3.22	0.004
	KDE_50_	0.09 ± 2.3	0.05 ± 0.7	2.32	0.021
Summer (Jun–Aug)	MCP	0.32 ± 2.1	0.24 ± 1.0	2.26	0.010
	KDE_95_	0.44 ± 2.2	0.37 ± 2.1	3.61	0.012
	KDE_50_	0.06 ± 3.1	0.02 ± 1.5	2.06	0.022
Autumn (Sep–Nov)	MCP	0.47 ± 3.2	0.29 ± 0.5	3.38	0.004
	KDE_95_	0.62 ± 1.4	0.56 ± 1.2	1.10	0.034
	KDE_50_	0.13 ± 0.3	0.06 ± 0.3	2.81	0.015
Winter (Dec–Feb)	MCP	0.28 ± 1.3	0.17 ± 3.0	2.32	0.001
	KDE_95_	0.31 ± 2.1	0.31 ± 0.6	0.49	0.064 *
	KDE_50_	0.05 ± 3.2	0.01 ± 1.0	1.46	0.010
Average	MCP	0.36 ± 0.6	0.23 ± 0.5	2.26	0.030
	KDE_95_	0.47 ± 1.5	0.39 ± 0.2	2.25	0.006
	KDE_50_	0.08 ± 2.1	0.03 ± 0.1	1.80	0.042

* α showing no significant difference at >0.05. Data are expressed as means with standard error.

**Table 2:** Seasonal pattern of nutria activity in the Macdo wetland.

Seasons	Trap Locations ± S.E.						*p*-Value	
	Twilight		Day		Night		M	F
	M	F	M	F	M	F		
Spring	28.2 ± 1.20	25.1 ± 10	15.2 ± 0.3	13.1 ± 0.43	20.1 ± 0.21	16.2 ± 0.60	0.12	0.11
Summer	31.1 ± 0.18	27.2 ± 0.27	19.1 ± 0.84	21.3 ± 0.16	32.6 ± 0.01	26.2 ± 0.11	0.61	0.32
Autumn	21.1 ± 0.16	17.6 ± 0.15	14.1 ± 0.17	13.1 ± 0.11	22.1 ± 11	17.6 ± 0.07	0.07	0.13
Winter	12.3 ± 0.31	12.6 ± 1.2	10.4 ± 0.27	9.1 ± 0.12	7.3 ± 0.21	8.4 ± 0.16	0.04 *	0.02 *
*p*-value	0.03 *	0.01 *	0.06	0.10	0.31	0.51		

M = Male, F = Female, and S.E. = Standard Error; * α showing significant difference at <0.05.

## Data Availability

All used data are included within the manuscript.

## Supplementary Materials

The following supporting information can be downloaded at: https://www.mdpi.com/article/10.3390/ani13101716/s1, Table S1: Morphological measurement of 24 nutria radio tracked in the Macdo wetland in 2015–2016.
