# Supplementary material for: Radio Tracking Reveals the Home Range and Activity Patterns of Nutria (Myocastor coypus) in the Macdo Wetland in South Korea
Source: Animals (Basel). 2023 May 22;13(10):1716. doi: 10.3390/ani13101716 (PMC10215913; doi:10.3390/ani13101716)
Supplement: Supplementary file 1 [file animals-13-01716-s001.zip › animals-2383019-Supplementary-file..pdf]

Table S1

Nutria ID	Sex	Body Mass (kg)	Head–Body Length (mm)	No. Locations
N1	Female	6.12	570	114
N2	Female	3.13	470	115
N3	Male	4.25	530	143
N4	Male	3.35	480	113
N5	Male	3.73	460	41
N6	Male	4.33	515	111
N7	Male	6.55	625	61
N8	Female	4.35	545	54
N9	Male	5.18	575	31
N10	Female	4.79	565	184
N11	Female	4.04	528	70
N12	Male	5.36	580	33
N13	Female	3.84	480	12
N14	Female	5.27	610	52
N15	Male	5.15	570	155
N16	Female	5.51	580	188
N17	Male	4.22	525	120
N18	Male	4.74	530	0
N19	Female	5.25	580	195
N20	Female	4.31	539	86
N21	Male	5.39	585	85
N22	Female	5.30	596	84
N23	Female	5.20	605	117
N24	Male	5.71	625	143
